# The association of Epstein‐Barr virus infection with CXCR3^+^ B‐cell development in multiple sclerosis: impact of immunotherapies

**DOI:** 10.1002/eji.202048739

**Published:** 2020-11-25

**Authors:** Jamie van Langelaar, Annet F. Wierenga‐Wolf, Johnny P.A. Samijn, Caroline J.M. Luijks, Theodora A. Siepman, Pieter A. van Doorn, Andrew Bell, Menno C. van Zelm, Joost Smolders, Marvin M. van Luijn

**Affiliations:** ^1^ Department of Immunology MS Center ErasMS Erasmus MC University Medical Center Rotterdam The Netherlands; ^2^ Department of Neurology MS Center ErasMS Erasmus MC University Medical Center Rotterdam The Netherlands; ^3^ Department of Neurology Maasstad Hospital Rotterdam The Netherlands; ^4^ Institute of Cancer and Genomic Sciences University of Birmingham Birmingham United Kingdom; ^5^ Department of Immunology and Pathology Monash University and Alfred Health Melbourne Australia; ^6^ Neuroimmunology Research group Netherlands Institute for Neuroscience Amsterdam The Netherlands

**Keywords:** EBV, memory B cells, multiple sclerosis, natalizumab, plasma cells

## Abstract

Epstein–Barr virus (EBV) infection of B cells is associated with increased multiple sclerosis (MS) susceptibility. Recently, we found that CXCR3‐expressing B cells preferentially infiltrate the CNS of MS patients. In chronic virus‐infected mice, these types of B cells are sustained and show increased antiviral responsiveness. How EBV persistence in B cells influences their development remains unclear. First, we analyzed ex vivo B‐cell subsets from MS patients who received autologous bone marrow transplantation (*n* = 9), which is often accompanied by EBV reactivation. The frequencies of nonclass‐switched and class‐switched memory B cells were reduced at 3–7 months, while only class‐switched B cells returned back to baseline at 24–36 months posttransplantation. At these time points, EBV DNA load positively correlated to the frequency of CXCR3^+^, and not CXCR4^+^ or CXCR5^+^, class‐switched B cells. Second, for CXCR3^+^ memory B cells trapped within the blood of MS patients treated with natalizumab (anti‐VLA‐4 antibody *n* = 15), latent EBV infection corresponded to enhanced in vitro formation of anti‐EBNA1 IgG‐secreting plasma cells under GC‐like conditions. These findings imply that EBV persistence in B cells potentiates brain‐homing and antibody‐producing CXCR3^+^ subsets in MS.

## Graphical abstract

Using blood samples from autologous BMT‐ and natalizumab‐treated MS patients, we demonstrate that high B‐cell EBV load associates with the development of CXCR3^+^ class‐switched B cells (ex vivo) and plasma cells under IL‐21‐, CD40L‐ and IFN‐γ‐inducing conditions (in vitro). CXCR3 expression on these plasma cells correlated with enhanced EBNA1‐IgG secretion.

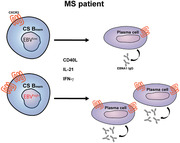

## Introduction

The success of anti‐CD20 therapy puts forward B cells as important players of autoimmune diseases including multiple sclerosis (MS) [1]. The strongest infectious risk factor for MS is the Epstein–Barr virus (EBV) [2, 3], a DNA γ‐herpes virus that infects and persists within B cells using a series of programs that mimic germinal center (GC) differentiation signals [4]. In MS, one of the hypotheses is that EBV remains latent within pathogenic memory B cells that invade the central nervous system (CNS) [3, 5]. Once in the CNS, pathogenic memory B cells are likely reactivated and further develop into antibody‐secreting plasma cells to mediate local inflammation [1]. IL‐21 and CD40L are the main triggers of plasma cell differentiation during a GC response [6, 7]. However, in murine models of autoimmune diseases this response seems to be dysregulated in the presence of IFN‐γ [8].

IFN‐γ triggering of B cells induces the T‐box transcription factor T‐bet, which drives the surface expression of CXC chemokine receptor (CXCR)3 [9]. Recently, our group found that CXCR3‐expressing B cells are abundant in the MS CNS and selectively accumulate in the blood of MS patients treated with natalizumab (NTZ; anti‐VLA‐4 mAb) [10], an effective drug that prevents their infiltration into the CNS. Moreover, naive B cells of untreated MS patients preferentially developed into CXCR3‐expressing plasmablasts under IL‐21‐, CD40L‐, and IFN‐γ‐inducing conditions in vitro [10]. Interestingly, in mice, CXCR3(T‐bet)‐expressing B cells do not only show increased antiviral responses, but are also more sustained during chronic viral infections [11]. How EBV infection contributes to the development of pathogenic memory B cells is poorly understood.

Here, we used two distinct MS cohorts to determine the association of B‐cell EBV DNA load with CXCR3^+^ memory B‐cell development. First, memory B‐cell subsets were studied ex vivo from MS patients who received autologous bone marrow transplantation (BMT), which is often accompanied with EBV reactivation [12, 13]. Second, GC‐like plasma cell differentiation was explored in vitro for memory B cells trapped within the blood of NTZ‐treated MS patients, who are known to experience a massive local influx of EBV‐infected B cells after treatment cessation [14].

## Results and discussion

### EBV load corresponds to CXCR3 expression in class‐switched memory B cells of BMT‐treated MS patients

**Figure 1:**
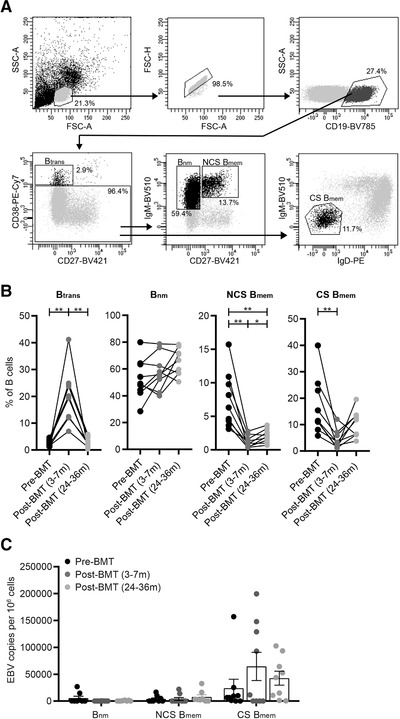
Reconstitution and EBV load of B‐cell subsets in the blood of autologous BMT‐treated MS patients. (A) Flow cytometry gating strategy used to define transitional (CD38^high^CD27^–^; B_trans_), naive mature (CD27 ^–^IgM^+^; B_nm_), nonclass‐switched memory (CD27^+^IgM^+^; NCS B_mem_), and class‐switched memory (IgM^–^IgD^–^; class‐switched [CS] B_mem_) within blood CD19^+^ B cells. (B) Quantification of B‐cell subset frequencies in the blood of MS patients before (black dots) and both 3–7 months (dark gray dots) and 24–36 months (light gray dots) after receiving autologous BMT (paired samples; *n* = 9; Supporting Information Table S1). (C) EBV DNA load in sorted B_nm_, NCS B_mem_, and CS B_mem_ cells before and both 3–7 and 24–36 months after BMT (*n* = 9; copies/1 × 10^6^ cells). Flow cytometry data were measured and B cell subsets were sorted in five independent experiments, with paired time points for one to two patients per experiment. EBV DNA load was determined by qPCR in three independent experiments, with paired time points for three to five patients per experiment. Data are presented as the mean ± SEM. **p < 0.01 and *p < 0.05. The *p* values in (B) were calculated by repeated measures one‐way ANOVA with Tukey's post hoc test.

**Figure 2:**
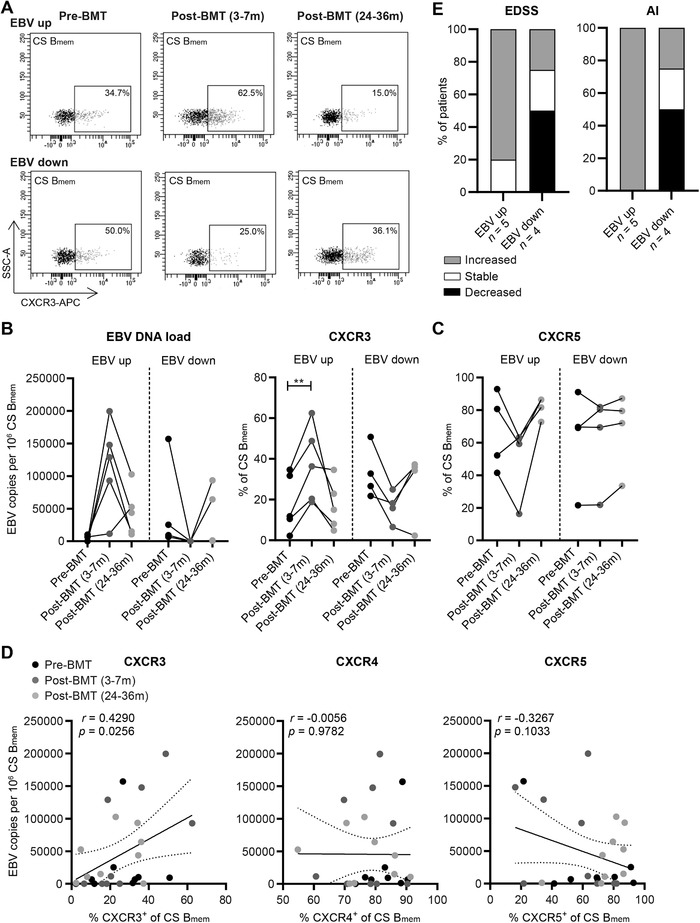
The association between EBV load and chemokine receptor expression in class‐switched B cells of BMT‐treated MS patients. (A) Representative flow cytometry plots of CXCR3‐expressing class‐switched (CS) B_mem_ cells from an MS patient before and both 3–7 months and 24–36 months after autologous BMT. EBV copy numbers measured by qPCR and frequencies of CXCR3^+^ (B) as well as CXCR5^+^ (C) fractions were determined for CS B_mem_ cells of nine BMT‐treated MS patients. (D) EBV copy numbers were correlated to CXCR3^+^, CXCR4^+^, and CXCR5^+^ fractions of CS B_mem_ cells. Data were collected in the same number of experiments as depicted in Figure 1. (E) Expanded disability status scale (EDSS) and ambulatory index (AI) changes after BMT treatment (∆ pre‐ vs. 36 months post‐BMT) for patients showing increased (*n* = 5) and decreased (*n* = 4) memory B‐cell EBV loads and measured by questionnaires. **p < 0.01. The *p* values were calculated by repeated measures one‐way ANOVA with Tukey's post hoc test (B and C) and correlation coefficient by Pearson rank (D).

To address the specific relation between EBV load and CXCR3^+^ B‐cell induction, we explored the distribution, EBV load, and chemokine receptor profiles of ex vivo B‐cell subsets within the blood of nine MS patients after autologous BMT (Supporting Information Table S1) [15]. The proportions of transitional (CD38^high^CD27^–^) B cells were increased at 3–7 months and reduced to baseline levels at 24–36 months, while those of naive mature (CD38^–/dim^IgM^+^CD27^–^) B cells remained unaltered post‐BMT (Fig. 1A and B). Both Ig class‐switched (IgM^–^IgD^–^) and nonclass‐switched (IgM^+^CD27^+^) memory B cells were decreased at 3–7 months, and only class‐switched B cells returned to baseline levels at 24–36 months post‐BMT (Fig. 1A and B). After purifying these subsets, high EBV copy numbers were measured for class‐switched B cells at 3–7 months post‐BMT (Fig. 1C), at the timeframe in which low numbers of memory B cells are present in the blood. This is in line with previous studies using allogeneic stem cell transplants [16, 17]. EBV levels in class‐switched B cells were increased for five patients and reduced for four patients when comparing 3–7 months post‐ to pre‐BMT samples. These changes in EBV load strongly corresponded to the expression of CXCR3 (Fig. 2A and B). Although immunomodulatory treatment was discontinued at least 1 month before BMT, six patients were previously treated with interferon‐beta (IFN‐β) or IFN‐β together with intravenous immunoglobulin (IVIG) [15]. Nevertheless, EBV load and CXCR3 expression in class‐switched B cells were similar to patients without previous treatment (Supporting Information Fig. S1 and Table S1). Taking all individual samples into account, we found a positive correlation between EBV load and CXCR3^+^, but not CXCR4^+^ or CXCR5^+^ fractions of class‐switched B cells (Fig. 2C and D). This was particularly significant for 3–7 months post‐BMT samples and not seen in pre‐BMT samples (Supporting Information Fig. S2A) or in blood samples from healthy controls (Supporting Information Fig. S2B). Despite the lower levels of EBV, similar trends were observed in nonclass‐switched B cells (Supporting Information Fig. S3A). In contrast to nonclass‐switched B cells, EBV load positively correlated to the expression of activation markers CD69 and CD95 in class‐switched B cells (Supporting Information Fig. S3B). Notably, three out of four BMT‐treated patients whose memory B cells showed decreased EBV and CXCR3 levels had stable or improved disability scores during 36 months follow‐up as reflected by expanded disability status scale score and ambulatory index (Fig. 2E) [15]. In BMT‐treated patients with an increase in memory B cell EBV and CXCR3 levels, an increased disability was observed in four out of five and five out of five participants, respectively.

Overall, these findings implicate that the high EBV levels found after autologous BMT in MS patients induces the development of CXCR3^+^ memory B cells and is possibly related to clinical worsening. CXCR3 upregulation through EBV infection may be caused by a disturbed immune system [18], and contribute to the increased recruitment of pathogenic B cells and the formation of follicle‐like structures in the MS CNS [10, 19].

### EBV^high^ memory B cells of NTZ‐treated MS patients preferentially develop into CXCR3^+^ plasma cells

**Figure 3:**
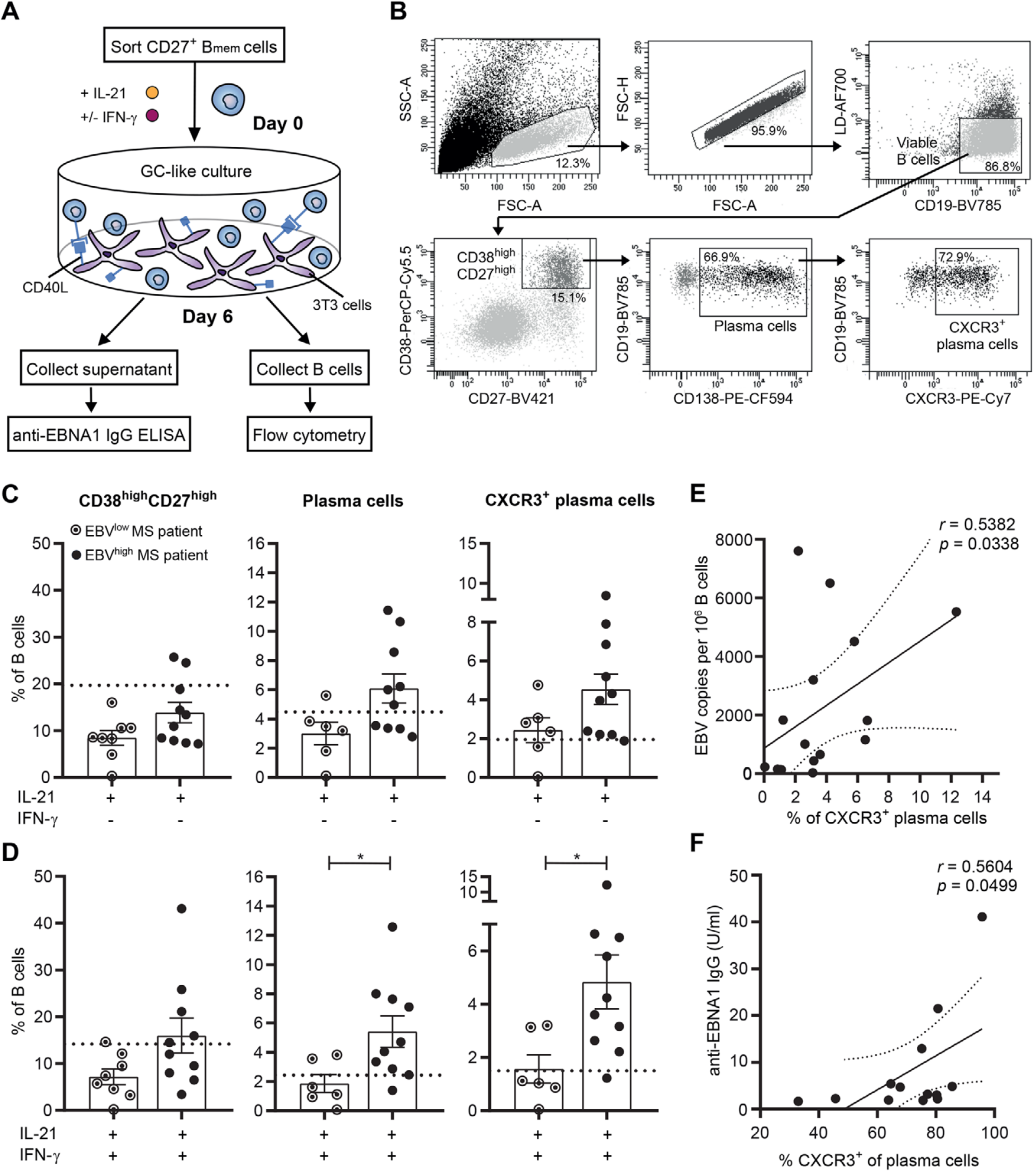
In vitro plasma cell formation of memory B cells from NTZ‐treated MS patients with different B‐cell EBV load. (A) Experimental setup of the GC‐like memory B‐cell differentiation assay. CD27^+^ memory B cells (B_mem_) were sorted from different blood samples of a total of 15 NTZ‐treated MS patients (Supporting Information Table S1) and cultured under IL‐21‐/CD40L‐inducing (GC‐like) conditions for 6 days. (B) Representative flow cytometry gating strategy for the analysis of in vitro differentiated plasma cells within viable CD19^+^ cells after 6 days of culturing. Plasma cell development was analyzed for NTZ‐treated MS patients with high and low B‐cell EBV load (*n* = 6–10) under conditions with and without IFN‐γ (C and D). Dotted lines indicate the mean frequencies of each population analyzed for simultaneous in vitro cultures with memory B cells of six age‐ and gender‐matched healthy controls. In vitro cultures and flow cytometry data were collected in six independent experiments, with one to two EBV^high^ and one to two EBV^low^ MS patients, as well as one healthy control per experiment. (E) Correlation between EBV copy numbers and fractions of in vitro induced CXCR3^+^ plasma cells. (F) Correlation between anti‐EBNA1 IgG secretion and CXCR3 surface expression by in vitro differentiated plasma cells. Anti‐EBNA1 IgG was measured in four independent experiments with two to six patient samples per experiment and each sample was measured in duplicate. Data are presented as the mean ± SEM. *p < 0.05. The *p* values were calculated by Mann–Whitney U (D) and correlation coefficients by Spearman rank (E and F) tests.

EBV mainly persists within GC‐derived memory B cells, which subsequently differentiate into antibody‐secreting plasma cells [4]. After NTZ treatment, MS patients show increased numbers of CXCR3^+^ memory B cells in the blood, which are normally recruited and further differentiate into plasma cells in the CNS [10, 20]. Hence, blood samples from these patients offer a unique opportunity to assess the relation between latent EBV infection and plasma cell development of potentially CNS‐infiltrating memory B cells. To determine this, we screened EBV load in different blood samples from 15 MS patients treated with NTZ for 1–4 years. Of these patients, one showed an increase, one a decrease, and the remainder a stable expanded disability status scale score over the course of 36 months after sampling (Supporting Information Table S1). Ten samples with >1000 copies per 1 × 10^6^ cells (EBV^high^) and eight samples with <500 copies per 1 × 10^6^ cells (EBV^low^) were selected for our in vitro differentiation study. CD27^+^ memory B cells were cultured under IL‐21‐ and CD40L‐stimulating (GC‐like) conditions for 6 days and differentiation into plasma cells (CD38^high^CD27^high^CD138^+^) was compared with and without the addition of IFN‐γ (Fig. 3A and B). Under these circumstances, total and CXCR3^+^ plasma cells were found to be more induced in the EBV^high^ versus the EBV^low^ group, which was most pronounced in cultures containing IFN‐γ (*p* = 0.023 and *p* = 0.011, respectively; Fig. 3C and D). For nine out of ten samples from the EBV^high^ group, CXCR3^+^ plasma cell frequencies were higher under IFN‐γ‐inducing conditions than those from a healthy reference group (*n* = 6; dotted line), which was the case for two out of six samples from the EBV^low^ group. B‐cell EBV load correlated to CXCR3^+^ plasma cell outgrowth in IL‐21‐ and IFN‐γ‐induced samples (Fig. 3E). This agrees with a mouse study showing that enhanced CXCR3 expression results in aberrant plasma cell development within GCs [9]. Both surface CXCR3 and intracellular T‐bet levels were further triggered by IFN‐γ during these cultures, but were not different between EBV^high^ and EBV^low^ groups (Supporting Information Fig. S4A). Finally, the ability of in vitro induced plasma cells to secrete anti‐EBV nuclear antigen 1 (anti‐EBNA1) IgG positively correlated with the expression of CXCR3 (Fig. 3F) and T‐bet (Supporting Information Fig. S4B).

## Concluding remarks

In this study, we reveal that high EBV load is associated with (1) early emergence of CXCR3^+^ class‐switched memory B cells from autologous BMT‐treated MS patients, and (2) enhanced in vitro generation of CXCR3^+^ plasma cells from memory B cells trapped in the blood of NTZ‐treated MS patients. Although we did not show the dependence of memory B‐cell development on CXCR3 in these settings, there is a clear relation between CXCR3 expression and GC‐like B‐cell development [9] and recruitment to the CNS [21] in virus‐infected mice. Based on our findings, EBV load is related to the development of CXCR3^+^ memory B cells into plasma cells, while the expression of CXCR3 by these plasma cells seems to enhance their ability to secrete anti‐EBNA1 IgG. This not only links to the predictive value and intrathecal detection of anti‐EBV antibodies [22, 23], but also contributes to the ongoing debate about the local presence of EBV‐infected B cells in MS [24]. CXCR3^+^ B cells may also strongly respond to other viruses implicated in MS [11, 25]. Subsequent studies should be performed to verify whether the impact of EBV load on CXCR3^+^ B‐cells is a general feature of MS regardless of disease‐modifying treatment.

## Materials and methods

### Patients

Characteristics of patients used in this study are summarized in Supporting Information Table S1. For the analysis of EBV infection in relation to ex vivo B‐cell subsets, we used nine MS patients who had undergone autologous BMT as previously described [15]. In short, CD34^+^ stem cells were isolated from BM and cryopreserved until use. These patients obtained a vigorous T‐cell depletion regimen consisting of ATG (anti‐thymocyte globulin) and total body irradiation before treatment with the preserved stem cells [15]. For each patient, thawed peripheral blood mononuclear cells (PBMCs) were analyzed before and both 3–7 and 24–36 months after BMT. Previous immunomodulatory drugs were discontinued at least 1 month prior to BMT (see also Supporting Information Fig. S1). ATG was given to patients 3–7 days before BMT [15]. PBMCs were isolated from six patients within 1 month and from three patients within 3 months before BMT. For all patients, PBMCs were collected before the start of ATG treatment. For the analysis of EBV load in relation to CXCR3^+^ B‐cell differentiation in vitro, we used thawed PBMCs of MS patients treated with NTZ (anti‐VLA‐4 antibody; 1–4 years posttreatment) [10]. These patients were both age‐ and gender‐matched with healthy controls. Study protocols were approved by the medical ethics committee of the Erasmus Medical Center (Rotterdam, The Netherlands) and all patients gave written informed consent [15, 26].

### Cell isolation, antibodies, and flow cytometry

PBMCs were isolated from blood of patients using Ficoll^®^‐Paque Plus (GE Healthcare, Freiburg, Germany) and density centrifugation or with vacutainer CPT^®^ tubes (BD Biosciences, Erembodegem, Belgium) and stored in liquid nitrogen until use as earlier reported [10]. FACS was performed on PBMCs using monoclonal antibodies as indicated in Supporting Information Table S2. Cells were stained extracellularly for 30 min at 4°C. Prior to extracellular staining, cultured memory B cells were labeled with a viability stain (FVS700) for the analysis of viable cells. All measurements were performed on a LSRII‐Fortessa machine and analyzed using FACS Diva software, version 8.0.1 (both BD Biosciences). B‐cell subsets were separated using a FACSAria III cell sorter (BD Biosciences) for qPCR analysis or in vitro cultures.

### DNA isolation and EBV load qPCR

DNA was extracted using a GenElute^TM^ Mammalian Genomic DNA Miniprep Kit according to the manufacturer's instructions (Sigma‐Aldrich via Merck, Kenilworth, NJ, USA). EBV DNA load was determined using a multiplex RQ‐PCR assay using a FAM‐labeled probe specific for *BALF5* (the EBV DNA polymerase; Eurogentec, Liège, Belgium) and a VIC‐labeled probe specific for reference gene beta‐2 microglobulin (*B2M*; Thermo Fisher Scientific, Landsmeer, The Netherlands) [27, 28]. Primer and probe sequences and used concentrations are shown in Supporting Information Table S3. We added 5 μL (200 ng) DNA to 20 μL reaction mixture containing primers and probes for *BALF5* and *B2M* supplemented with TaqMan^TM^ Universal Master Mix II, no UNG (Applied Biosystems^TM^, Thermo Fisher Scientific). Samples were run on a QuantStudio^TM^ 5 machine (Applied Biosystems^TM^, Thermo Fisher Scientific) using the following thermal cycle protocol: 10 min at 95°C followed by 50 cycles of 15 s at 95°C and 1 min at 60°C.

EBV genome copies per 1 × 10^6^ cells were calculated using standard curves for *BALF5* and *B2M* as analyzed and generated by the QuantStudio^TM^ Design and Analysis software version 1.4.1. The standard curves were made by serial dilutions of Namalwa DNA [27] containing 40^4^ (stock concentration: 132 ng/μL DNA; assuming that 1 cell contains 6.6 pg DNA and each Namalwa cell contains two integrated EBV genomes), 10^4^, 10^3^, 200, 40, 10, 4, and 1 EBV genome per μL H_2_O. The quantity of EBV DNA in sorted B cells was extrapolated from these curves using CT values measured in each experiment. All samples were measured in duplicate.

### In vitro memory B‐cell differentiation assay

Sorted CD27^+^ memory B cells were cultured under GC‐like conditions as previously described [10]. In short, irradiated 3T3‐CD40L cells were seeded on flat‐bottom 96‐well plates. We added 5 × 10^4^ memory B cells per well in the presence of human recombinant IL‐21 (50 ng/mL; Thermo Fisher Scientific) with and without human recombinant IFN‐γ (50 ng/mL; Peprotech/Bio‐Connect, Huissen, The Netherlands). After 6 days of culture, supernatants were collected and stored at −80℃ until use for an EBNA‐1 IgG enzyme‐linked immunosorbent assay (ELISA). The cultured cells were removed and extracellularly stained using flow cytometry as described above.

### Anti‐EBNA1 IgG ELISA

A commercially available ELISA kit (IBL international, Hamburg, Germany) was used to quantify anti‐EBNA‐1 IgG antibodies in memory B‐cell culture supernatants. Note that 50 μL of each supplied standard and culture supernatants were added to the plate and incubated for 60 min at room temperature (RT). After washing, samples were incubated with rabbit peroxidase‐conjugated IgG for 30 min at RT. Each well was incubated with TMB substrate solution for 20 min at RT and the reaction was stopped with TMB solution. Optical densities were measured at 450 nm using a BioTek Synergy 2 machine (Winooski, Vermont, USA). Data were analyzed by following the manufacturer's instructions. A cut‐off value for culture supernatants was determined based on the average from cultured naive mature B cells (*n* = 8). Results above ≥1.59 U/mL were considered positive, which was the case for 13 out of 21 samples.

### Statistical analysis

Statistical analyses on datasets were carried out using GraphPad Prism Software, version 8 (GraphPad Software, San Diego, CA) and indicated in figure legends. Data are depicted as the mean ± standard error of the mean or 95% confidence intervals for correlations. Probability values < 0.05 were considered significant.

## Author contributions

J.v.L., M.M.v.L., A.B., and M.C.v.Z. contributed to study concept and design. J.P.A.S. and P.A.v.D. collected and supplied autologous BMT patient material. J.v.L., A.F.W., C.J.M.L., and T.A.S. acquired and analyzed data. J.v.L., J.S., and M.M.v.L. drafted the manuscript and figures.

### Peer review

The peer review history for this article is available at https://publons.com/publon/10.1002/eji.202048739.

## Conflict of Interest

J.S. received speaker/consultancy fee from Biogen, Merck, Novartis, and Sanofi‐Genzyme. All other authors have no commercial or financial conflicts of interest.

**Abbreviations**
BMT: bone marrow transplantation
EBNA1: EBV nuclear antigen 1
IVIG: intravenous immunoglobulin
NTZ: natalizumab
RT: room temperature

## Supporting information

Supporting Information

## Data Availability

The data that support the findings in this study are available from the corresponding author upon reasonable request.
