# Supplementary material for: The association of Epstein‐Barr virus infection with CXCR3+ B‐cell development in multiple sclerosis: impact of immunotherapies
Source: Eur J Immunol. 2020 Nov 25;51(3):626–33. doi: 10.1002/eji.202048739 (PMC7984177; doi:10.1002/eji.202048739)
Supplement: Supplementary file 1 — Supporting Information [file EJI-51-626-s001.pdf]

## Supplementary Table 1. Demographics of MS patients included in this study

| *Ex vivo* B-cell analysis | | | | | | | | |
|---|---|---|---|---|---|---|---|---|
| **BMT-treated MS patient[a]** | **Age (years)[b]** | **Gender** | **Disease duration (months)[c]** | **Previous treatment** | **EDSS** | | **AI** | |
| | | | | | **Pre-BMT** | **36m Post-BMT** | **Pre-BMT** | **36m post-BMT** |
| 1 | 47 | M | 60 | - | 6.5 | 6.5 | 4 | 6 |
| 2 | 44 | F | 48 | IFN-β | 6.5 | 8 | 6 | 9 |
| 3 | 31 | F | 60 | IFN-β | 5.5 | 4.5 | 4 | 3 |
| 4 | 37 | F | 60 | - | 6 | 5.5 | 4 | 4 |
| 5 | 50 | F | 48 | - | 5.5 | 6.5 | 3 | 6 |
| 6 | 41 | F | 24 | IFN-β + IVIG | 6 | 6 | 6 | 5 |
| 7 | 23 | M | 72 | IFN-β + IVIG | 6 | 8 | 5 | 9 |
| 8 | 34 | F | 36 | IFN-β | 5 | 6.5 | 3 | 6 |
| 9 | 34 | M | 144 | IFN-β | 6 | 7 | 4 | 7 |

| *In vitro* B-cell analysis | | | | | | | |
|---|---|---|---|---|---|---|---|
| **NTZ-treated MS patient** | **Age (years)[b]** | **Gender** | **Disease duration (months)[c]** | **Previous treatment** | **EDSS** | | **AI** |
| | | | | | **Pre-NTZ** | **36m post-NTZ** | |
| 1 | 41 | F | 44 | NA | 3.5 | 3.5 | NA |
| 2 | 38 | M | 41 | NA | 3.5 | 3.5 | NA |
| 3 | 36 | F | 104 | NA | 4 | 4 | NA |
| 4 | 46 | F | 46 | NA | 3 | 3 | NA |
| 5 | 44 | F | 5 | NA | 3.5 | 3.5 | NA |
| 6 | 46 | M | 88 | NA | 3.5 | 3.5 | NA |
| 7 | 21 | M | 39 | NA | 2.5 | 2.5 | NA |
| 8 | 29 | M | 124 | NA | 4.5 | 4.5 | NA |
| 9 | 33 | F | 131 | NA | 6.5 | 6.5 | NA |
| 10 | 25 | F | 79 | NA | 1.5 | NA[d] | NA |
| 11 | 37 | F | 109 | NA | 6.5 | 6.5 | NA |
| 12 | 34 | F | 45 | NA | 2.5 | 2.5 | NA |
| 13 | 49 | F | 192 | NA | 5 | 4 | NA |
| 14 | 27 | M | 2 | NA | 4.5 | 4.5 | NA |
| 15 | 28 | M | 11 | NA | 2.5 | 4 | NA |

[a]Secondary progressive MS patients who had received an autologous bone marrow transplantation [15]

[b]At start of treatment

[c]Time from MS diagnosis to start of treatment

[d]This patient discontinued natalizumab at 24 months of treatment

BMT = bone marrow transplant; MS = multiple sclerosis; IFN-β = interferon-beta; IVIG = intravenous immunoglobulin; EDSS = expanded disability status score; AI = ambulatory index; NTZ = natalizumab; NA = not applicable

## Supplementary Figure 1

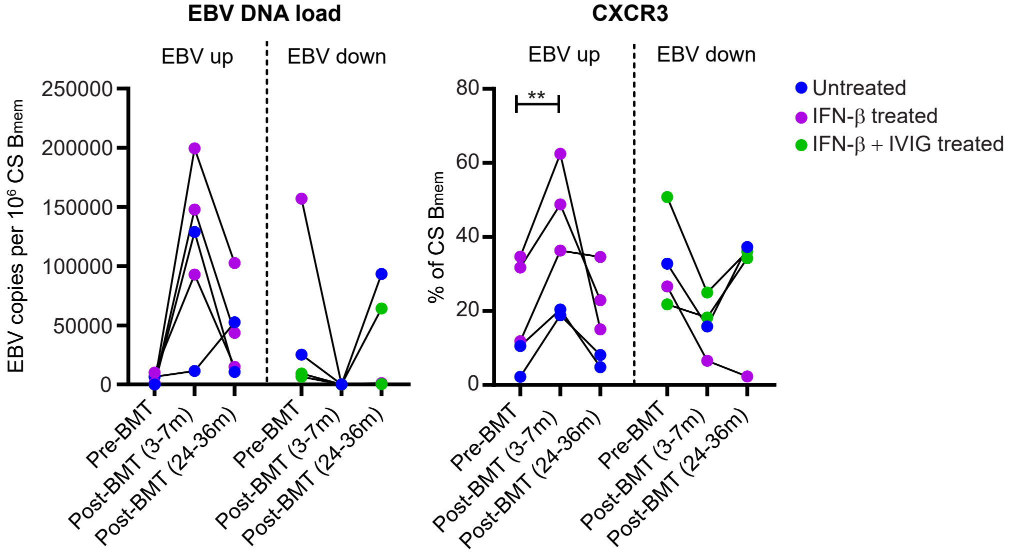


**Supplementary Figure 1. Immunomodulatory treatment prior to BMT has no effect on EBV load or CXCR3 expression in CS $B_{mem}$ cells.**

EBV copy numbers and frequencies of $CXCR3^{+}$ fractions were determined for CS $B_{mem}$ cells of 9 BMT-treated MS patients. Treatment given more than 1 month prior to BMT: interferon beta (IFN-β; purple, $n$ = 4) and IFN-β + intravenous immunoglobulin (IVIG; green $n$ = 2). Data were collected in the same number of experiments as depicted in Figure 1. ** $p$ <0.01.

**Supplementary Figure 2**

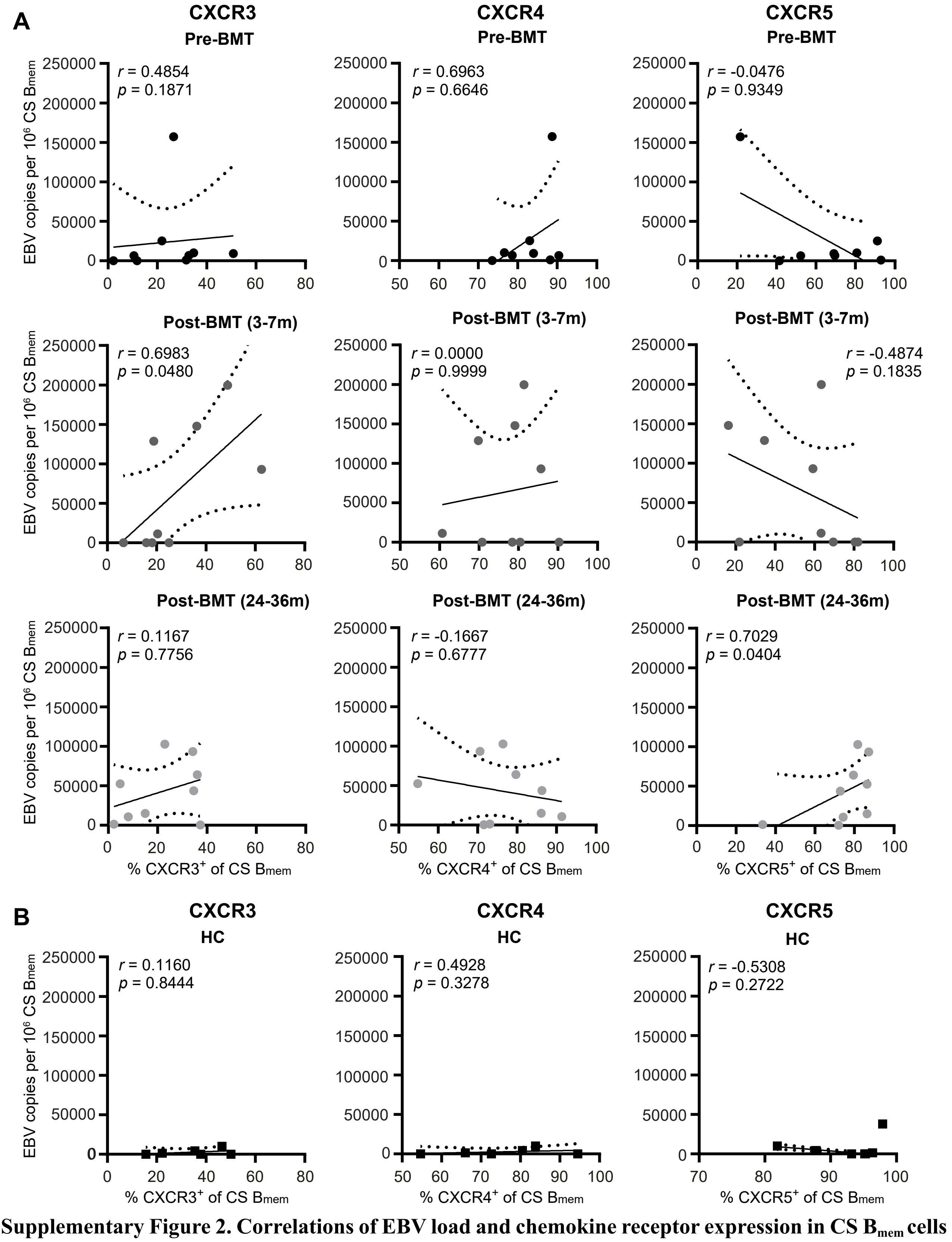


**Supplementary Figure 2. Correlations of EBV load and chemokine receptor expression in CS $B_{mem}$ cells in BMT-treated MS patients and healthy controls.**

EBV copy numbers were correlated to CXCR3, CXCR4 and CXCR5 surface expression in CS $B_{mem}$ cells of (**A**) pre-BMT, 3-7 months post- and 24-36 months post-BMT (*n* = 9 in each case) and (**B**) healthy controls (*n* = 6). Data were collected in the same number of experiments as depicted in Figure 1. The *p* values and correlation coefficients were calculated by Spearman rank test.

Supplementary Figure 3

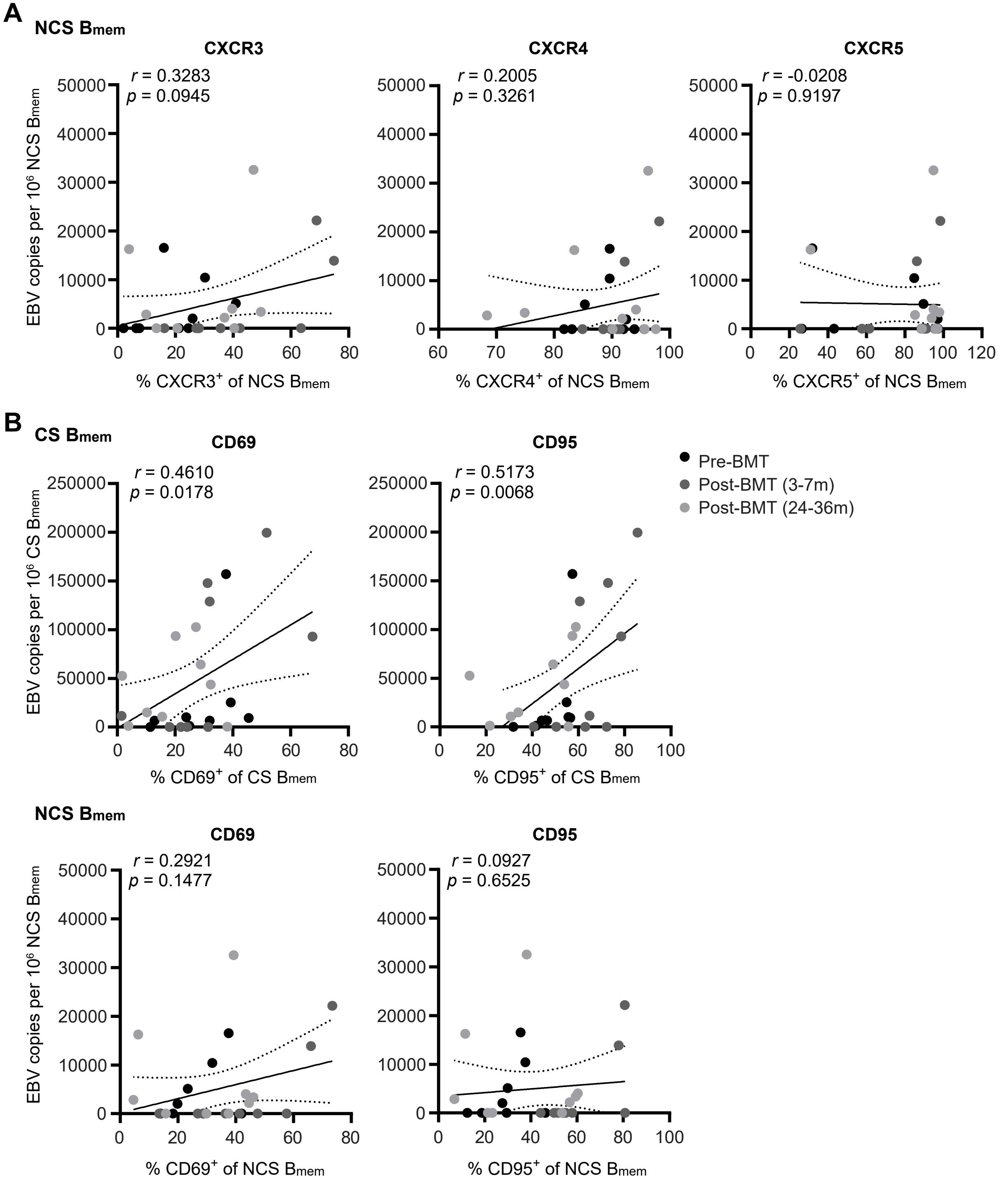


**Supplementary Figure 3. Correlations of EBV load to chemokine receptor expressions on NCS $B_{mem}$ cells and activation markers on CS and NCS $B_{mem}$ cells.**

EBV copy numbers were correlated to CXCR3, CXCR4 and CXCR5 surface expression in NCS Bmem cells (**A**), and to surface expression of CD69 and CD95 in both CS and NCS $B_{mem}$ cells (**B**) of 9 BMT-treated MS patients. Data were collected in the same number of experiments as depicted in Figure 1. The *p* values and correlation coefficients were calculated by Pearson rank test.

## Supplementary Figure 4

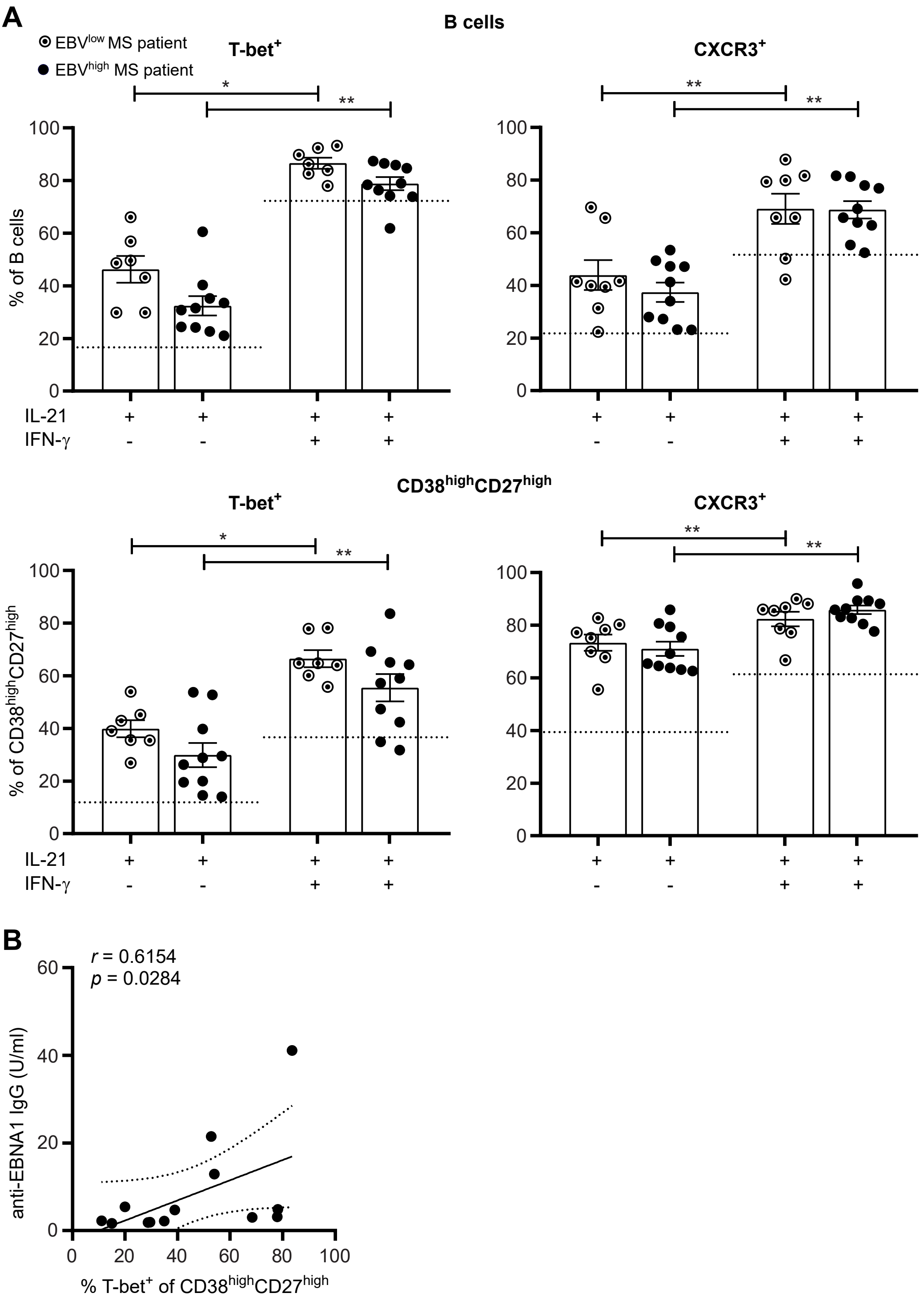


**Supplementary Figure 4. T-bet and CXCR3 expression is induced by IFN-γ and anti-EBNA1 IgG levels correlate to T-bet expression in $CD38^{high}CD27^{high}$ cells during *in vitro* cultures.**

(**A**) Surface expression of T-bet and CXCR3 on total $CD19^{+}$ B cells and $CD38^{high}CD27^{high}$ cells were analyzed for NTZ-treated MS patients with high ($n$ = 10) and low ($n$ = 7) B-cell EBV load cultured under GC-like conditions with and without IFN-γ. Dotted lines indicate the mean frequencies of T-bet and CXCR3 analyzed for age-/gender-matched healthy controls ($n$ = 6). (**B**) Correlation between anti-EBNA1 IgG and the surface expression of T-bet on $CD38^{high}CD27^{high}$ cells. Data were collected in the same number of experiments as depicted in Figure 3. Data are presented as the mean ± SEM. $^{**}p$ <0.01 and $^{*}p$ <0.05. The $p$ values were calculated by Wilcoxon matched-pairs signed rank (**A**) and correlation coefficients by Spearman rank (**B**) tests.

## Supplementary Table 2. Monoclonal antibodies used for FACS analysis

| Antibody Marker | Fluorochrome | Clone | Company |
|---|---|---|---|
| CD3 | FITC | SK7 | BD Biosciences |
| CD19 | BV785 | HIB19 | BD Biosciences |
| CD27 | BV421 | M-T271 | BD Biosciences |
| CD38 | PE-Cy7 and PerCP-Cy5.5 | HIT2 | Biolegend |
| CD69 | APC-R700 | FN50 | BD Biosciences |
| CD95 | BV605 | DX2 | Biolegend |
| CD138 | PE-CF594 | MI15 | BD Biosciences |
| CXCR3 | BV605, APC, PE-Cy7 | G025H7 | Biolegend |
| CXCR4 | PE-CF594 | 12G5 | BD Biosciences |
| CXCR5 | PerCP-Cy5.5 | RF8B2 | BD Biosciences |
| IgD | PE | IA6-2 | BD Biosciences |
| IgM | BV510 | MHM-88 | Biolegend |
| Fixable viability dye | AF700 | | BD Biosciences |

**Supplementary Table 3. Primers and probes used for qPCR**

| | |
|---|---|
| **BALF5** | |
| Forward primer (10 µM) | CTTTGGCGCGGATCCTC |
| Reverse primer (10 µM) | AGTCCTTCTTGGCTAGTCTGTTGAC |
| Probe (5 µM) | (FAM)-CATCAAGAAGCTGCTGGCGGCC-(TAMRA) |
| **B2M** | |
| Forward primer (3 µM) | GGAATTGATTTGGGAGAGCATC |
| Reverse primer (4 µM) | CAGGTCCTGGCTCTACAATTTACTAA |
| Probe (5 µM) | (VIC)-AGTGTGACTGGGCAGATCATCCACCTTC-(TAMRA) |
